# Pain: A Distributed Brain Information Network?

**DOI:** 10.1371/journal.pbio.1002037

**Published:** 2015-01-06

**Authors:** Hiroaki Mano, Ben Seymour

**Affiliations:** 1 Center for Information and Neural Networks, National Institute for Information and Communications Technology, Osaka, Japan; 2 Immunology Frontiers Research Center, Osaka University, Suita, Japan; 3 Computational and Biological Learning Laboratory, Department of Engineering, University of Cambridge, Cambridge, United Kingdom

## Abstract

Understanding how pain is processed in the brain has been an enduring puzzle, because there doesn't appear to be a single “pain cortex” that directly codes the subjective perception of pain. An emerging concept is that, instead, pain might emerge from the coordinated activity of an integrated brain network. In support of this view, Woo and colleagues present evidence that distinct brain networks support the subjective changes in pain that result from nociceptive input and self-directed cognitive modulation. This evidence for the sensitivity of distinct neural subsystems to different aspects of pain opens up the way to more formal computational network theories of pain.

## Abstract 2

How is pain processed in the brain? This Primer discusses evidence that the nociceptive input and cognitive modulation are processed independently as parts of an integrated brain network from which subjective pain emerges. See the Research Article.

On the surface, pain should have been one of the easier brain systems to understand. Its fundamental importance in organism defence means that its anatomy should be well conserved across species, unlike systems for language, for instance. And its relatively simple scalar signal (from less pain to more pain) should not require extensive computational processing, unlike sound or vision. However, since Penfield's failure to convincingly locate a “pain cortex” during his classic awake brain stimulation studies in the 1950s [1], trying to piece together the pain system in the brain has been a story of frustration and debate.

## The Missing Pain Cortex

Anatomically, the problem starts in the peripheral nerve and spinal cord. There are two broadly different types of nociceptor (“slow burning” c-fibers and “fast sharp” a-delta fibers), which feed into several ascending spinal pathways heading towards the brain [2]. These spinal pathways serve not only thalamic targets (both medial and lateral) but also a number of key brainstem nuclei, such as the parabrachial nucleus and periaqueductal grey, each of which also project higher in the brain [3],[4]. With so many roads to the cortex, therefore, it's clear that teasing apart distinct brain functions might not be so straightforward.

In the face of this complexity, a very reasonable approach is to try to work backwards from subjective pain experience and behaviour. However, reverse engineering the pain system from behavioural studies has not been easy, something that is easy to understand when one considers the multidimensional nature of the feeling of pain. Introspection alone illustrates seemingly distinct sensory (the “what and where” of pain), emotional (“how much it hurts”), and cognitive (“attention-grabbing”) characteristics of pain, famously outlined in Wall and Melzack's tripartite model [5]. Indeed, this ultimately phenomenological dissociation has stuck, in the absence of any better theory, and has provided an almost universally adopted framework for the interrogation of human functional neuroimaging studies that have been possible since the advent of positron emission tomography (PET) and functional magnetic resonance imaging (fMRI).

However, brain mapping of pain has come up against two problems. The first relates to the inherently subjective quality of pain. Hence, psychological manipulations have tended to focus on dissociation on individual ratings of intensity (“sensory dimension”) or unpleasantness (“emotional dimension”)—components that are not always as readily distinguishable to the experimental subject as they are to the scientist. Notwithstanding this, there was an early hope that the sensory and emotional dimensions might be easily mapped to two relatively distinct streams of cortical processing, named the medial (emotional) and lateral (sensory) pain systems [6]. Although this offered reassuring parallels with other sensory brain systems, such as the ventral and dorsal visual streams, this distinction has not endured as well, largely due to a second, much bigger problem: functional multiplicity.

The functional multiplicity problem refers to the fact that many brain regions, especially those typically activated by pain, seem to end up being activated by quite a lot of other things as well [7]. This wasn′t widely appreciated in the early days of neuroimaging and led to a culture of “reverse inference” that was pervasive across many domains of cognitive science [8],[9]. For example, because pain activated regions such as the anterior cingulate cortex, it was often wrongly assumed that other tasks which activate the anterior cingulate must be psychologically “painful” in some manner [10]. This difficulty in defining functional anatomy arises from two possible causes: either the macroscale resolution of fMRI is not able to adequately resolve heterogenous microscale neuronal processing units (anatomy), or we haven′t defined the psychological processes (functions) correctly.

The latter possibility feeds the hypothesis that subjectively unitary dimensions of pain might actually be constructed not by separate feed-forward processing streams, but by a distributed set of interacting functional units, or even multiple independent pain modules. Such a parallel and reciprocally connected architecture negates neither regional functional specialization nor processing hierarchies, but emphasizes the known bidirectional network structure of pain, with cooperating ascending and descending pathways from dorsal horn to cortex. But how we read information from this distributed anatomy involving (currently) functionally obscure modules creates a new problem, and one in which practical solutions have only recently emerged.

## Probing the Pain Matrix as an Information Network

Multivariate pattern analysis (or “brain decoding”) approaches to functional neuroimaging allow interrogation of information arising from multiple sites. In contrast to mass univariate approaches that directly map a (voxel-by-voxel) signal in one region to an experimental variable, multivariate approaches allow us relate information from multiple regions to behaviour. This approach was first applied to decode aspects of subjective visual perception from fMRI data from the visual cortex, where it was shown that features could be “decoded” that weren′t obvious to conventional approaches [11]. In 2010, it was shown that such an approach could also be applied to predict subjective pain experience from activity across the whole brain [12]. However, perhaps the most robust demonstration to date came from a study from a recent paper by Wager and colleagues, who demonstrated a highly accurate and specific mapping from brain to experienced pain, and critically distinguishes physical pain from its neural impersonators [13]. They termed this “decoder” the neurological pain signature (NPS).

The NPS basically represents a specific pattern of activity across the many brain regions—perhaps not surprisingly, many of these turn out to be those that are typically associated with pain in conventional studies (often called the “pain matrix”) [14]. Since the pattern incorporates potentially complex codependences and interactions between these areas, it isn't a pattern that can be easily seen by the naked eye, but instead it is a core pattern of the information that distinguishes pain from non-pain. Whereas this might sound satisfying for an information scientist, it is not immediately clear what this means in terms of neurophysiological mechanisms.

## Modulating the Pain Network

One way to try and get more insight into the biological mechanisms underlying any multivariate pattern is to go back and carefully perform behavioural studies. In their study reported in this issue of *PLOS Biology*, Woo and colleagues consider whether or not the NPS is influenced by psychological procedures that typically modulate pain. They used a cognitive regulation paradigm to amplify or reduce pain—a conscious process where subjects rethink their pain using mental imagery and self-instruction, following which they receive various levels of experimental heat pain. This is a classic “top-down” modulatory paradigm, and it works very well to modulate the experience of phasic pain. The key question is then what happens to the NPS and whether it tracks the modulated pain.

**Figure 1:**
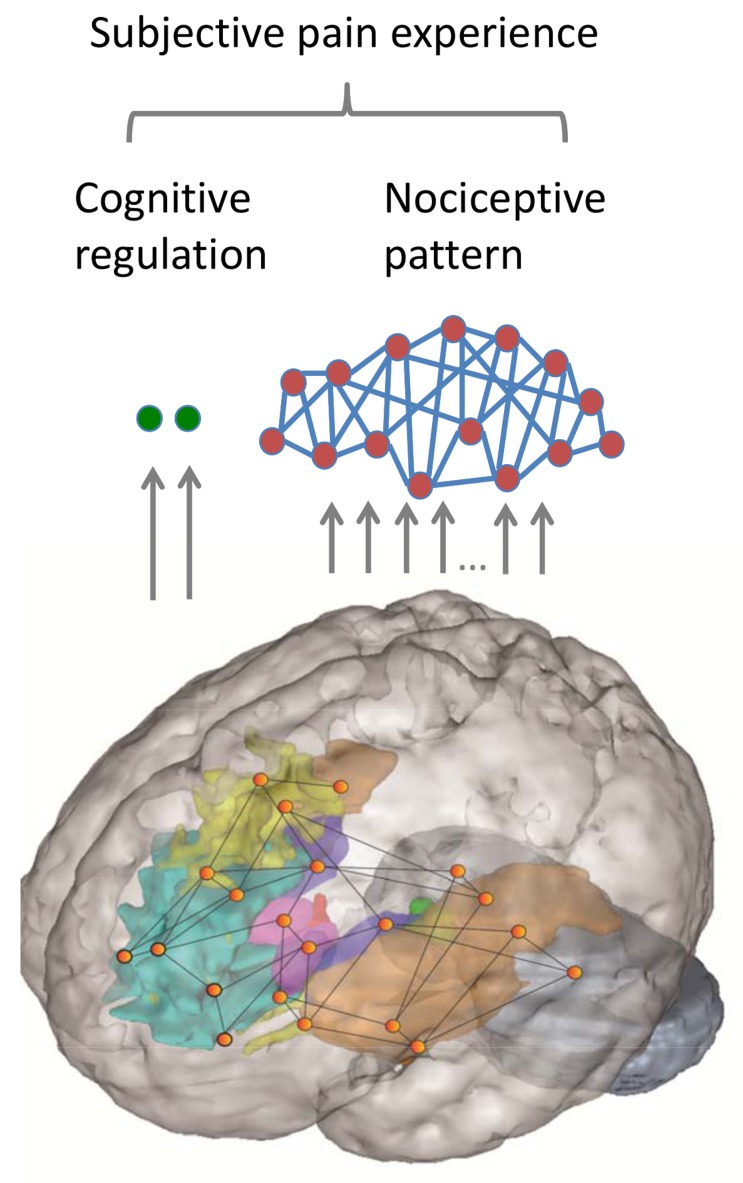
Distinct component to the subjective perception of pain. Core nociceptive nodes comprise a multivariate pattern (the neurological pain signature [NPS]), and fronto-striatal brain regions comprise an evaluative pathway sensitive to self-directed cognitive modulation.

Remarkably, it seems to be almost completely unaffected. This means that whatever information is used in the NPS decoding, it doesn't simply represent the subjective experience of pain. Instead, the authors found that the influence of modulation is reflected in different brain regions—notably the nucleus accumbens and ventromedial prefrontal cortex (in brief, greater activity reflects less pain). So perhaps in this study the NPS just doesn't work, and instead nucleus accumbens and ventromedial prefrontal cortex (vmPFC) reflect the true subjective experience of pain? Not so—because they also separately modulated the intensity of nociceptive stimulation: this turns out to control the NPS, but has no effect on the accumbens–vmPFC axis. In summary, although both modulation and stimulus intensity change subjective pain experience, they seem to do so by entirely separate, non-interacting processes (Fig 1).

Some might be tempted to seize upon this result as actually a “failure” of the currently popular multivariate pattern analysis approach—because clearly the NPS doesn't capture the entirety of subjective pain. But more likely it simply adds weight to the notion that subjective pain might not be reducible to activity of a single brain region, but reflects some coordinated activity within a network of functionally distinct regions. According to this view, subjective pain emerges from a distributed brain network, as opposed to a single “pain cortex.” However, we should cautiously note that there is still some wriggle room for a theory of a single subjective pain region because it is entirely possible that such an area exists that is simply not discernible by current neuroimaging technology.

Can we go further and propose that independent networks can independently cause pain? This may be premature because we don′t know whether there is a pattern of brain activity that subsumes all types of pain and pain modulation. If there is, the key question is whether the network topology that generates this pattern is defined by interactions between regions (network “nodes”) that comprise independent or near-independent processing modules. This will turn out to be an important direction for future research, especially with regards to understanding the nature of other types of modulation, such as the placebo effect. It may be that a much faster timescale resolution (than is permissible with fMRI) will be required to capture dynamic network properties. However, linear decoding (such as the NPS) and potentially more complicated machine learning methods (such as deep learning) represent a potentially valuable information theoretic approach to probe the relationship between phasic (experimental) and chronic (clinical) pain [15]. In the case of the latter, these results are important to understand the theoretical limitation on efficacy of treatments of pain that target single brain areas, such as deep brain stimulation and neurofeedback [16].

## Pain and Reward

The results cast a spotlight on the role of the nucleus accumbens and vmPFC in pain, as these areas are not classically considered to have a dominant role in pain. Instead, these regions are typically associated with reward, so what might be their role be in cognitive pain modulation? The vmPFC is best understood to play a role in reward-based decision-making, in which it is thought to code the values of putative goal-directed actions [17]. Here, however, unless the activity represents the intrinsic instrumental reward of successful self-regulation (i.e., satisfying the experimental instructions), there is no clear action–reward contingency. Instead, it might be more likely to represent the value of pain relief. Several studies of avoidance learning and reward–avoidance interactions have shown that avoidance values are also coded in vmPFC [18]–[21], so this seems potentially more plausible (note that this may not be in the same neurons as reward: there is a longstanding debate about whether safety and reward share functional equivalence) [22],[23]. This could difficult to prove because of the experimental difficulty of controlling internally motivated goals (i.e., self-regulation).

If the cognitive regulation was a purely instrumental strategy, we might also expect to see dorsal striatal activity. Instead, the authors identify activity in the (anterior) ventral striatum (i.e., nucleus accumbens), which is more commonly associated with passive (Pavlovian) reward prediction. One explanation is that in this context the accumbens activity is not a reward prediction signal at all (dopaminergic reward “wanting”), but a hedonic reward signal (putatively opioidergic reward “liking”) [24]. Accordingly to Fields' influential account of reward–pain interactions—the Motivation-Decision model [25]—such an appetitive hedonic representation can directly inhibit pain, and so the nucleus accumbens would be a prime candidate to implement such a mechanism. Ultimately, however, this leads to the question of what determines the subjective nature of pleasantness and whether it might share a distributed network representation, as postulated for pain.

## From Information to Computation

Whatever the mechanisms, the results demonstrate how using an information-driven approach leads to computational questions about the mechanisms of pain modulation. There is a genuinely valid debate to be aimed at data-driven analysis methods such as multivariate pattern analysis, in that these methods don't clearly test neurophysiological hypotheses and don't uncover the mechanisms of behaviour. This debate sits within the much broader tension between data-driven and hypothesis-driven research, which has always been a contentious issue since the early days of neural networks and currently is manifest with the popularity of “big data.” Woo and colleagues' study illustrates an intelligent way forward. Given that pain is a poorly understood, complex brain system, it is reasonable to integrate a data-driven approach to identify the nature and structure of information in broad brain networks, in parallel with asking more specific hypothesis-driven questions about the functional operations that the individual regions in those networks perform.
